# Quantitative Multiparametric MRI as an Imaging Biomarker for the Prediction of Breast Cancer Receptor Status and Molecular Subtypes

**DOI:** 10.3389/fonc.2021.628824

**Published:** 2021-09-16

**Authors:** Zhiqi Yang, Xiaofeng Chen, Tianhui Zhang, Fengyan Cheng, Yuting Liao, Xiangguan Chen, Zhuozhi Dai, Weixiong Fan

**Affiliations:** ^1^Department of Radiology, Meizhou People’s Hospital, Meizhou, China; ^2^Guangdong Provincial Key Laboratory of Precision Medicine and Clinical Translational Research of Hakka Population, Meizhou, China; ^3^Pharmaceutical Diagnostics, GE Healthcare, Guangzhou, China; ^4^Department of Radiology, Shantou Central Hospital, Shantou, China

**Keywords:** molecular subtypes, receptor status, breast neoplasms, pharmacokinetics, diffusion weighted imaging, magnetic resonance imaging, dynamic contrast enhanced magnetic resonance imaging (DCE-MRI)

## Abstract

**Objectives:**

To assess breast cancer receptor status and molecular subtypes by using the CAIPIRINHA-Dixon-TWIST-VIBE and readout-segmented echo-planar diffusion weighted imaging techniques.

**Methods:**

A total of 165 breast cancer patients were retrospectively recruited. Patient age, estrogen receptor, progesterone receptor, human epidermal growth factorreceptor-2 (HER-2) status, and the Ki-67 proliferation index were collected for analysis. Quantitative parameters (K^trans^, V_e_, K_ep_), semiquantitative parameters (W_-in_, W_-out_, TTP), and apparent diffusion coefficient (ADC) values were compared in relation to breast cancer receptor status and molecular subtypes. Statistical analysis were performed to compare the parameters in the receptor status and molecular subtype groups.Multivariate analysis was performed to explore confounder-adjusted associations, and receiver operating characteristic curve analysis was used to assess the classification performance and calculate thresholds.

**Results:**

Younger age (<49.5 years, odds ratio (OR) =0.95, *P*=0.004), lower K_ep_ (<0.704,OR=0.14, *P*=0.044),and higher TTP (>0.629 min, OR=24.65, *P*=0.011) were independently associated with progesterone receptor positivity. A higher TTP (>0.585 min, OR=28.19, *P*=0.01) was independently associated with estrogen receptor positivity. Higher K_ep_ (>0.892, OR=11.6, *P*=0.047), lower TTP (<0.582 min, OR<0.001, *P*=0.004), and lower ADC (<0.719 ×10^-3^ mm^2^/s, OR<0.001, *P*=0.048) had stronger independent associations with triple-negative breast cancer (TNBC) compared to luminal A, and those parameters could differentiate TNBC from luminal A with the highest AUC of 0.811.

**Conclusions:**

K_ep_ and TTP were independently associated with hormone receptor status. In addition, the K_ep_, TTP, and ADC values had stronger independent associations with TNBC than with luminal A and could be used as imaging biomarkers for differentiate TNBC from Luminal A.

## Introduction

Breast cancer (BC) is the most common cancer diagnosed in women and has a high mortality (1). Molecular subtypes of BC, based on genotype variations, are critical in determining treatments and predicting prognosis (2–4). Due to the high cost of full genetic analysis, the immunohistochemical (IHC) surrogate biomarkers, including estrogen receptor (ER), progesterone receptor (PR), human epidermal growth factor receptor-2 (HER-2), and the Ki-67proliferation index, are routinely used to define these subtypes, which are major prognostic factors in guiding targeted therapy and predicting tumor response to neoadjuvant chemotherapy (NAC) (5, 6). The current diagnosis of receptor status together with the Ki-67 proliferation index based on IHC requires tissue specimens obtained by invasive biopsy. Moreover, the biopsy and surgical specimens may have different receptor statuses and Ki-67 proliferation and these indicators may change during treatment (5). Therefore, more defined imaging parameters are needed to delineate these prognostic factors (7).

Recent studies have shown that multiparametric MRI features can distinguish benign breast tumors from malignant tumors (8, 9). Moreover, some reports have suggested that apparent diffusion coefficient (ADC) values are independently associated with triple-negative breast cancer (TNBC) and could be used to differentiate TNBC from luminal tumors (10). Wu et al. found that the imaging features of dynamic contrast-enhanced MRI (DCE-MRI) were associated with distinct molecular pathways (11). However, due to the limitations of low temporal resolution for conventional DCE-MRI and a low signal-to-noise ratio for conventional diffusion-weighted imaging (DWI), quantitative DCE-MRI and DWI analysis have not been routinely adopted in clinical practice.

Recently, CAIPIRINHA-Dixon-TWIST-VIBE (CDT-VIBE) has been shown to improve the temporal resolution of DCE-MRI with preserved spatial resolution by filling the k-space with aspiral orbi (9, 12) and is considered a potential candidate for conventional DCE-MRI. Readout-segmented echo-planar diffusion-weighted imaging (RS-EPI) can improve the signal-to-noise ratio and is considered a potential candidate for conventional DWI, demonstrating promising results in the abdomen (13, 14). However, the high-temporal resolution quantitative parameters from CDT-VIBE DCE-MRI and the diffusion parameters from RS-EPI DWI have not been studied for their ability to predict BC receptor status and molecular subtypes. Therefore, the aim of this study was to evaluate whether high-temporal resolution quantitative parameters and diffusion parameters can be used to predict BC receptor status and molecular subtypes by using CDT-VIBE and RS-EPI techniques.

## Materials and Methods

### Patients

**Figure 1:**
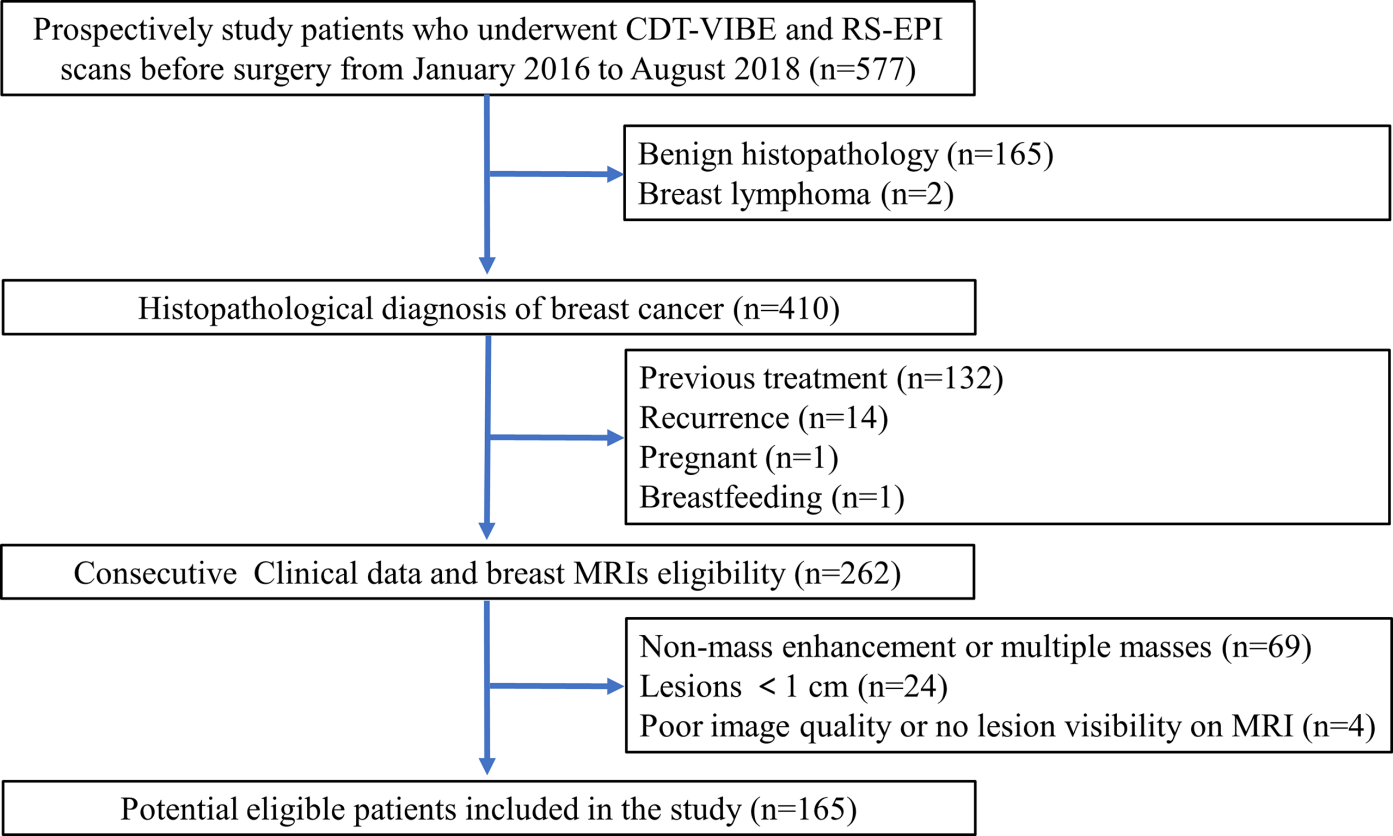
Patient selection flow chart.

This study obtained institutional review board approval, and the need for informed patient consent was waived for this retrospective study. A prospectively enrolled patients in our institution that consisted of 577 consecutive patients who underwent CDT-VIBE DCE-MRI and RS-EPI DWI of the breast between January 2016 and August 2018 was queried for patients who fulfilled the following inclusion criteria: postoperative histopathology confirmed BC with receptor status, no recurrence, no previous treatment, not pregnant, and not breastfeeding. A total of 262 consecutive patients matched our search criteria. The exclusion criteria were as follows: (1) nonmass enhancement or multiple masses; (2) lesions< 1.0 cm; and (3) poor image quality or no lesion visible on MRI. The patient selection is outlined in **Figure 1**.

### Immunohistochemical and Molecular Subtypes

IHC techniques were introduced to classify the different receptor statuses and molecular subtypes of BC. ER- and PR-positive tumors were defined as ≥1% positively stained tumor cells (15). HER2-positive tumors were defined as tumors with IHC staining of 3+ or gene amplification using fluorescence *in situ* hybridization demonstrated gene amplification >2.0 of tumors when IHC staining of 2+. Tumors with Ki-67≥20% were considered high Ki-67 expression tumors (16). According to the expression status of ER, PR, HER-2 and Ki-67, BC can be divided into four subtypes: luminal A, luminal B, HER-2 enriched, and TNBC. Luminal A was positive for ER and/or PR, Ki-67< 20% and HER-2-negative. Luminal B was ER- and/or PR-positive, HER-2-positive, or HER-2-negative when Ki-67 was ≥20%. HER-2 enrichment was ER- and PR-negative with HER-2 positivity. TNBC was all negative for ER, PR, and HER-2 (16).

### MRI Scanning

All examinations were performed on a 3.0 T MRI scanner (Magnetom Skyra, Siemens, Germany) using a 16-channel bilateral breast coil with the patient positioned in the center of the magnet in the prone position. The MRI protocols included CDT-VIBE DCE-MRI, RS-EPI DWI, and conventional T_1_-and T_2_-weighted imaging. The RS-EPI sequence was performed prior to DCE-MRI. The parameters of the CDT-VIBE sequence (80-85 slices) were as follows: TR/TE = 6.4/3.3 ms, FA = 9°, FOV = 288 mm × 384 mm, matrix =448×314, bandwidth = 870 Hz,4 factor CAIPIRINHA acceleration, 2 mm slice thickness without slice gap. A total of 34 phases with a temporal resolution of 8.7 s were obtained within 5 min 5 s, starting 17.7 s after intravenous injection of 0.2 ml/kg of gadopentetate dimeglumine (Bayer Pharma AG) at an injection flow rate of 3.0 ml/s. An RS-EPI sequence (50-55 slices) with 2 b-values (50, 800 s/mm^2^) (TR/TE = 4800/56 ms, FA = 180°, FOV = 170 mm × 340 mm, matrix=96×192, bandwidth = 868 Hz, 4 mm slice thickness with 0.8 slice gap, and a total acquisition time of 4 min 58 s) was performed. T_1_-weighted acquisition (TR = 5.5 ms, TE = 2.5 ms, FOV = 341 mm×341 mm, slice thickness = 1.5 mm, slice gap = 0.3 mm, bandwidth = 430 Hz, total acquisition time = 1 min 40 s) and T_2_-weighted acquisition (TR = 3570 ms, TE = 74 ms, FOV = 341 mm×341 mm, slice thickness = 1.5 mm, slice gap = 0.3 mm, bandwidth = 248 Hz, total acquisition time =2 min 23 s) was also performed.

### Image Evaluation

MRI images were independently evaluated by two senior radiologists with more than ten years of breast MRI experience. They were both blinded to the patients’ clinical history and histopathological results. DCE-MRI, DWI and ADC maps from each patient were transferred to the Siemens Syngo workstation. DCE-derived parametric maps of quantitative and semiquantitative parameters were automatically generated after motion correction, and registration was performed by using Tissue 4D software tool (Syngo *via* VB10, Siemens Healthcare).The pharmacokinetic parameters (17), including the volume transfer constant (K^trans^, min^-1^), extravascular-extracellular space volume ratio (V_e_, min^-1^), rate constant (K_ep_=K^trans^/V^e^), rate of contrast enhancement for contrast agent inflow (W_-in_, min^-1^), rate of contrast decay for contrast agent outflow (W_-out_, min^-1^) and time-to-peak enhancement after contrast agent injection (TTP, min),were assessed with parametric maps by using the conventional Tofts model (9) and were then generated for each voxel defined by the region of interest (ROI). The RS-EPI DWI image (b= 800 s/mm^2^) and ADC maps were opened as read-only files, and the ADC value (×10 ^-3^mm ^2^/s) for the RS-EPI DWI was measured in the same region of the lesion as the DCE-derived parametric maps. The breast tumors were identified on DCE-MRI images with the prominent area of enhancement corresponding to the hyperintense regions on DWI (b=800 s/mm^2^) and the hypointense regions on ADC maps. One 2D-ROI with a minimum area of 10 mm^2^ was placed on the greatest representative slice of the tumor, avoiding obvious bleeding, visible necrosis, vessels, calcifications, and cystic appearing areas. The mean values were recorded after 3 repeated measurements.

To validate the stability of the measured parameters, Reader 1 performed tumor measurements in all 165 patients, and Reader 2 performed tumor measurements in 111 patients who were randomly selected from the whole cohort to assess interreader agreement. Only those parameters with an interclass correlation coefficient (ICC) value greater than 0.75 were selected for further experiments (18).

### Statistical Analysis

The statistical analysis was performed with R (version 3.5.1). The Mann–Whitney *U* test and *t*-test were used to compare the quantitative MRI parameters between the different receptor expression statuses. The Kruskal–Wallis *H* test and one-way ANOVA test were performed for different molecular subtypes.Multivariate analysis was performed to explore confounder adjusted associations among quantitative parameters, receptor status and molecular subtypes. We included all factors that were significant (*P*<0.05) upon univariate analysis. In addition, receiver operating characteristic (ROC) curve analysis was also carried out, and the optimal cutoff values of the quantitative parameters to predict receptor status and molecular subtype were determined according to the largest Youden index. The area under the curve (AUC), accuracy, sensitivity and specificity were also calculated.A *P* value <0.05 was considered to be statistically significant.

## Results

### Basic Clinicopathological Characteristics

A total of 165 patients were included in this study, and the mean patient age was 50 years (range:25~81 years). Most had invasive ductal carcinoma (150 lesions), and there were five ductal carcinomas *in situ*, two medullary carcinomas, one adenosquamous carcinoma and seven invasive lobular carcinomas. The table of patient’s characteristics are presented in **Table 1**. PR-positive patients were younger than PR-negative patients (*P*<0.01).

**Table 1:** MRI parameters of different receptor statuses in breast cancer.

Receptor status	ER		*P* value	PR		*P* value	HER-2		*P*-value	Ki-67		*P* value
	N (n = 61)	P (n = 104)		N (n = 97)	P (n = 68)		N (n = 111)	P (n = 54)		L (n = 43)	H (n = 122)	
Age^#^	51.5 ± 9.46	49.5 ± 11.4	0.241^a^	52.1 ± 10.5	47.5 ± 10.6	**0.007** ^a^	50.6 ± 11.2	49.6 ± 9.76	0.593^a^	50.7 ± 10.6	50.1 ± 10.8	0.757^a^
K^trans^	0.16 (0.09, 0.24)	0.13 (0.09, 0.26)	0.906^b^	0.15 (0.09, 0.27)	0.13 (0.09, 0.22)	0.637^b^	0.14 (0.09, 0.25)	0.14 (0.09, 0.28)	0.471^b^	0.14 (0.10, 0.21)	0.15 (0.09, 0.27)	0.779^b^
K_ep_	0.90 (0.76, 1.02)	0.82 (0.66, 0.96)	**0.010^b^**	0.90 (0.76, 1.02)	0.75 (0.63, 0.92)	**<0.001^b^**	0.82 (0.67, 0.96)	0.92 (0.75, 1.03)	**0.039^b^**	0.82 (0.65, 0.96)	0.88 (0.71, 0.98)	0.097^b^
V_e_	0.19 (0.09, 0.29)	0.21 (0.12, 0.31)	0.267^b^	0.19 (0.10, 0.29)	0.21 (0.13, 0.32)	0.292^b^	0.20 (0.12, 0.29)	0.18 (0.10, 0.32)	0.924^b^	0.19 (0.13, 0.26)	0.20 (0.10, 0.32)	0.778^b^
W_-in_	0.59 (0.48, 0.74)	0.57 (0.40, 0.77)	0.372^b^	0.60 (0.48, 0.76)	0.54 (0.39, 0.75)	0.123^b^	0.59 (0.42, 0.73)	0.58 (0.48, 0.79)	0.240^b^	0.49 (0.38, 0.84)	0.59 (0.47, 0.74)	0.371^b^
W_-out_	-0.01 (-0.03, -0.01)	-0.02 (-0.03, -0.00)	0.581^b^	-0.02 (-0.03, -0.01)	-0.01 (-0.02, 0.00)	**0.018^b^**	-0.01 (-0.02, -0.01)	-0.02 (-0.03, -0.01)	0.162^b^	-0.01 (-0.03, 0.00)	-0.02 (-0.03, -0.01)	0.145^b^
TTP	0.56 (0.50, 0.63)	0.62 (0.56, 0.77)	**0.001^b^**	0.58 (0.51, 0.64)	0.64 (0.57, 0.81)	**<0.001^b^**	0.59 (0.52, 0.75)	0.60 (0.53, 0.70)	1.000^b^	0.64 (0.52, 0.81)	0.59 (0.53, 0.70)	0.148^b^
ADC^#^	0.84 ± 0.13	0.83 ± 0.11	0.646^a^	0.82 ± 0.12	0.84 ± 0.12	0.217^a^	0.81 ± 0.11	0.88 ± 0.11	**<0.001^a^**	0.85 ± 0.12	0.82 ± 0.11	0.152^a^

^#^Indicates that the results are the mean value with standard deviation. All other results are the median with interquartile range in parentheses. P^a^: t-test. P^b^: Mann–Whitney U test.

N, Negative; P, Positive; H, High proliferating; L, Low proliferating; ER, Estrogen receptor; PR, Progesterone receptor; HER-2, Human epidermal growth factor receptor-2; ADC, Apparent diffusion coefﬁcient. Bold values indicate statistically significant in P values.

### Interobserver Agreement of Quantitative MRI Parameters

The ICC values of K^trans^, K_ep_, V_e_, TTP, W_-in_, W_-out_, and ADC were 0.962 (95% CI: 0.946-0.974), 0.874 (95% CI: 0.822-0.912), 0.919 (95% CI: 0.884-0.944), 0.924 (95% CI: 0.891-0.947),0.878 (95% CI: 0.826-0.914), 0.901 (95% CI: 0.855-0.933), and 0.797 (95% CI: 0.718-0.856), respectively. All the ICC values were greater than the threshold value of 0.75.

### Receptor Status

**Figure 2:**
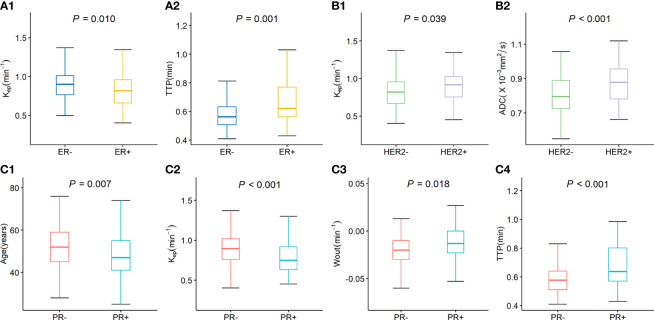
Box plot graphs revealing statistically significant differences in values for different receptor statuses. The K_ep_ and TTP values were significantly difference in ER status **(A1, A2)**. The K_ep_ and ADC values were significantly difference in HER-2 status **(B1, B2)**; The age, K_ep_ W-out, and TTP values were significantly difference in PR status **(C1–C4)**.

The quantitative parameters of DCE-MRI and ADC for tumors stratified by receptor status are summarized in **Table 1**. The K_ep_ values were lower when ER and PR were positive (all *P*<0.05). In contrast, the TTP value was higher when ER and PR were positive (all *P*<0.01). The K_ep_ and ADC values were higher when HER-2 was positive (all *P*<0.05).The W_-out_ value was higher when PR was positive (*P*<0.05). Box plot graphs revealing statistically significant differences in values for different receptor statuses are shown in **Figure 2**. The parameters K^trans^, W_-in_, and TTP were not significantly different in the expression statuses of ER, PR, HER-2 or Ki-67 (all *P*>0.05).

According to the univariate analysis (**Table 2**), a lower K_ep_ value (<0.704, OR=0.19, *P*=0.019) and a higher TTP value (> 0.585 min, OR=47.73, *P*=0.002) were significantly associated with ER-positive status. Younger age (<49.5 years, OR=0.96, *P*=0.008), a lower K_ep_ value (<0.704,OR=0.08, *P*=0.001), a higher W_-out_ value (<-0.005 min^-1^,OR=13.5, *P*=0.031) and a higher TTP value (> 0.629 min, OR=41.9, *P*<0.001) were significantly associated with PR-positive status. A higher ADC value (> 0.757×10^−3^mm^2^/s, OR=215.9, *P*<0.001) was significantly associated with HER-2-positive status. In the multivariate analysis, a higher TTP value (adjusted OR=28.19, *P*=0.01) remained independently associated with ER-positive status, while younger age (adjusted OR=0.95, *P*=0.004), a lower K_ep_ value (adjusted OR=0.14, *P* =0.044), and a higher TTP value (adjusted OR=24.62, *P* =0.011) remained independently associated with PR-positive status.

**Table 2:** Univariate and multivariate analysis of the parameters for different receptor statuses in breast cancer.

Receptor status	ER (negative *vs*. positive)					PR (negative *vs*. positive)					HER-2 (negative *vs*. positive)			Ki-67 (negative *vs*. positive)		
	Univariate analysis			Multivariate analysis		Univariate analysis			Multivariate analysis		Univariate analysis			Univariate analysis		
	OR	*P* value	cutoff	OR	*P* value	OR	*P* value	cutoff	OR	*P* value	OR	*P* value	cutoff	OR	*P* value	cutoff
Age	0.98	0.263	39.50	NA	NA	0.96	**0.008**	49.5	0.95	**0.004**	0.99	0.590	55.5	0.99	0.755	50.50
K^trans^	1.12	0.938	0.159	NA	NA	0.22	0.309	0.215	NA	NA	3.22	0.427	0.282	6.66	0.268	0.258
K_ep_	0.19	**0.019**	0.704	0.36	0.168	0.08	**0.001**	0.704	0.14	**0.044**	3.01	0.117	0.915	4.30	0.073	0.693
V_e_	4.43	0.268	0.096	NA	NA	3.26	0.358	0.112	NA	NA	1.03	0.981	0.182	0.97	0.985	0.275
W_-in_	0.59	0.455	0.410	NA	NA	0.38	0.174	0.425	NA	NA	3.05	0.125	0.605	1.35	0.700	0.462
W_-out_	12.48	0.762	-0.035	NA	NA	13.5	**0.031**	-0.005	0.06	0.807	<0.001	0.094	-0.025	<0.001	0.089	0.009
TTP	47.73	**0.002**	0.585	28.19	**0.01**	41.9	**<0.001**	0.629	24.65	**0.011**	0.44	0.450	0.562	0.18	0.121	0.635
ADC	0.53	0.643	0.861	NA	NA	5.48	0.217	0.927	NA	NA	215.9	**<0.001**	0.757	0.11	0.153	0.933

OR, Odds ratio; NA, Not available; ER, Estrogen receptor; PR, Progesterone receptor; HER-2, Human epidermal growth factor receptor-2; ADC, Apparent diffusion coefficient. Bold values indicate statistically significant in P values.

### Molecular Subtypes

**Table 3** shows the values of the quantitative parameters for different molecular subtypes. We found that K_ep_ was the highest in HER-2-enriched cells and the lowest in luminal A cells (*P*<0.05). TTP was the highest in luminal A and the lowest in TNBC (*P*<0.01). ADC was the highest in HER-2-enriched cells and the lowest in TNBC (*P*<0.001).

**Table 3:** MRI parameters for different molecular subtypes in breast cancer.

Subtype	Luminal A (n = 30)	Luminal B (n = 74)	HER-2-enriched (n = 27)	TNBC (n = 34)	*P* value
Age^#^	51.9 ± 11.4	48.5 ± 11.3	52.0 ± 9.50	51.0 ± 9.54	0.319^a^
K^trans^	0.15 (0.10, 0.22)	0.13 (0.09, 0.27)	0.15 (0.09, 0.22)	0.16 (0.09, 0.26)	0.991^b^
K_ep_	0.76 (0.62, 0.89)	0.86 (0.66, 0.98)	0.92 (0.76, 1.10)	0.90 (0.77, 0.99)	**0.036^b^**
V_e_	0.20 (0.13, 0.27)	0.21 (0.11, 0.32)	0.16 (0.09, 0.29)	0.19 (0.10, 0.29)	0.710^b^
W_-in_	0.52 (0.36, 0.82)	0.58 (0.44, 0.76)	0.56 (0.48, 0.76)	0.60 (0.48, 0.74)	0.689^b^
W_-out_	-0.01 (-0.03, 0.00)	-0.02 (-0.03, -0.01)	-0.02 (-0.03, -0.01)	-0.01 (-0.02, -0.01)	0.490^b^
TTP	0.70 (0.58, 0.86)	0.61 (0.56, 0.74)	0.60 (0.51, 0.71)	0.55 (0.49, 0.58)	**0.001^b^**
ADC^#^	0.85 ± 0.10	0.82 ± 0.12	0.90 ± 0.11	0.78 ± 0.11	**<0.001^a^**

^#^Indicates the results are the mean value with standard deviation. All other results are the median with interquartile range in parentheses. P^a^: one-way ANOVA test. P^b^: Kruskal–Wallis H test. TNBC, Triple-negative breast cancer; ADC, Apparent diffusion coefﬁcient. Bold values indicate statistically significant in P values.

**Figure 3:**
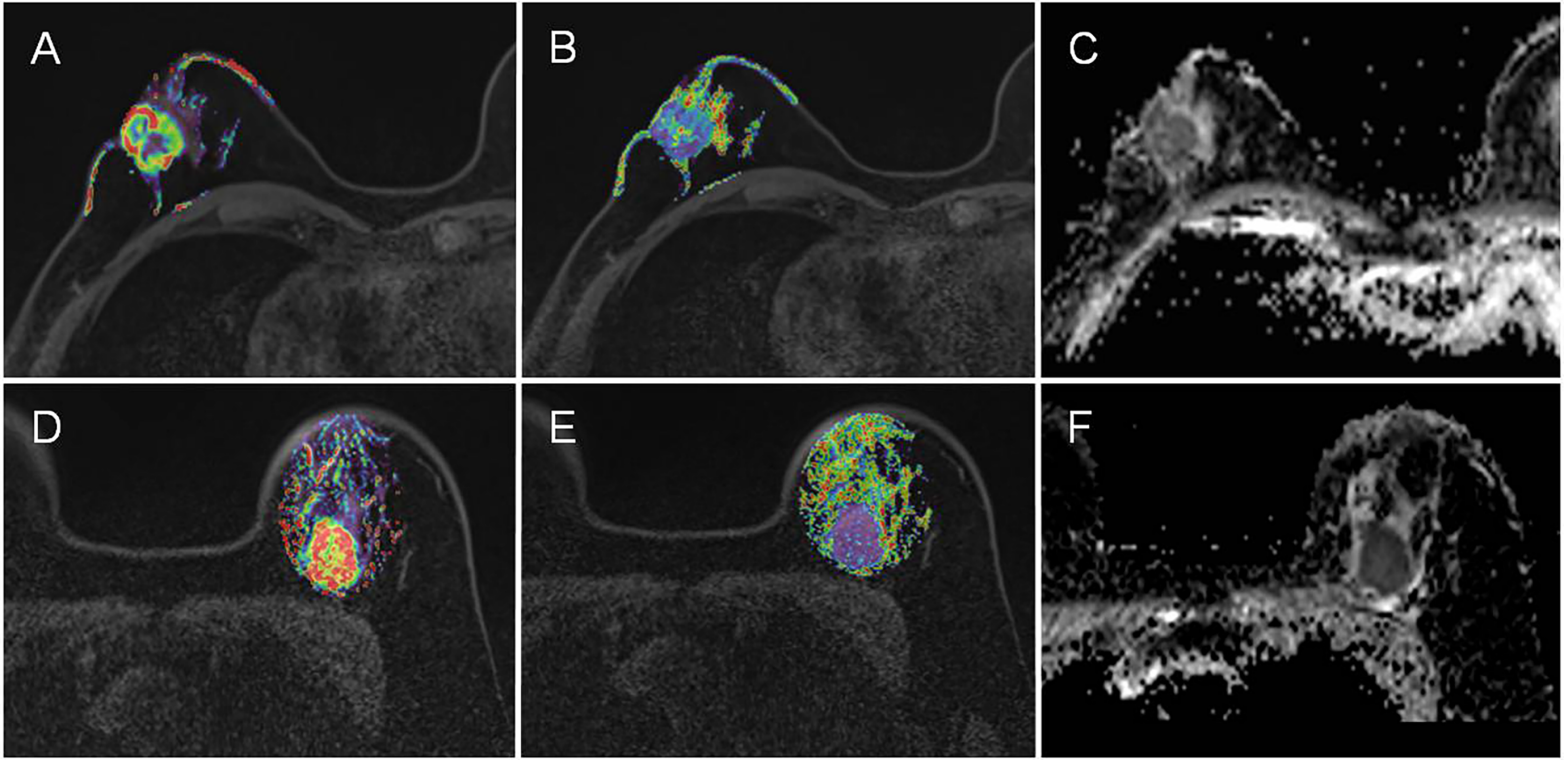
Representative K_ep_, TTP and ADC images in luminal A and TNBC. **(A–C)** are the images of K_ep_, TTP and ADC from a luminal A patient, with mean values of 0.48 min^-1^, 0.70 min, and 0.89×10^-3^ mm^2^/s, respectively. **(D–F)** are the images of K_ep_, TTP and ADC from a TNBC patient, with mean values of 0.94 min^-1,^ 0.58 min, and 0.70×10^-3^ mm^2^/s, respectively.

**Table 4** shows the pairwise comparison analysis of the parameters of different subtypes. Further paired comparisons revealed that the K_ep_ values were significantly different between luminal A and HER-2 enrichment and between luminal A and TNBC (all *P*<0.05), but only the difference between luminal A and TNBC was confirmed in the univariate analysis (*P*<0.05). The TTP value was significantly different between luminal A and TNBC, between luminal B and TNBC, and between HER-2-enriched BC and TNBC (all *P*< 0.05), and those differences were confirmed in the univariate analysis (all *P*<0.05).The ADC value was significantly different between luminal A and TNBC, between luminal B and HER-2-enriched BC, and between HER-2-enriched BC and TNBC (all *P*<0.05), and those differences were confirmed in the univariate analysis (all *P*<0.05). In the multivariate analysis lower TTP (<0.582 min, adjusted OR<0.001,*P* =0.004) and lower ADC values (<0.719×10^-3^ mm^2^/s, adjusted OR<0.001,*P* =0.048) were significantly associated with TNBC compared to luminal A; a lower ADC value (<0.759 ×10^-3^ mm^2^/s, adjusted OR<0.001, *P* =0.002) was significantly associated with TNBC compared to HER-2 enrichment. Representative K_ep_, TTP and ADC images in luminal A and TNBC are shown in **Figure 3**.

**Table 4:** Ppairwise comparison analysis of the parameters in different subtypes.

		Mann–Whitney *U* or *t*-test	Univariate analysis		Multivariate analysis	
Comparative groups	Parameters	*P* value	OR	*P* value	OR	*P* value
Luminal A *vs.* Luminal B	K_ep_	0.169^b^	2.98	0.251	NA	NA
	TTP	0.148^b^	0.16	0.149	NA	NA
	ADC	0.145^a^	0.06	0.146	NA	NA
Luminal A *vs.* HER-2-enriched	K_ep_	**0.041^b^**	10.9	0.050	NA	NA
	TTP	0.080^b^	0.04	0.072	NA	NA
	ADC	0.088^a^	85.2	0.092	NA	NA
Luminal A *vs.* TNBC	K_ep_	**0.016^b^**	11.6	**0.047**	4.67	0.202
	TTP	**<0.001^b^**	<0.001	**<0.001**	<0.001	**0.004**
	ADC	**0.014^a^**	<0.001	**0.018**	<0.001	**0.048**
Luminal B *vs.* HER-2-enriched	K_ep_	0.137^b^	4.36	0.139	NA	NA
	TTP	0.527^b^	0.30	0.427	NA	NA
	ADC	**0.002^a^**	570	**0.003**	NA	NA
Luminal B *vs.* TNBC	K_ep_	0.154^b^	3.93	0.131	NA	NA
	TTP	**0.001^b^**	<0.001	**0.003**	NA	NA
	ADC	0.173^a^	0.08	0.173	NA	NA
HER-2-enriched *vs.* TNBC	K_ep_	0.738^b^	0.92	0.947	NA	NA
	TTP	**0.049^b^**	0.01	**0.039**	0.03	0.163
	ADC	**<0.001^a^**	<0.001	**0.001**	<0.001	**0.002**

P^a^: t-test. P^b^: Mann–Whitney U test. OR, Odds ratio; NA, Not available; TNBC, Triple-negative breast cancer; ADC, Apparent diffusion coefﬁcient. Bold values indicate statistically significant in P values.

**Figure 4:**
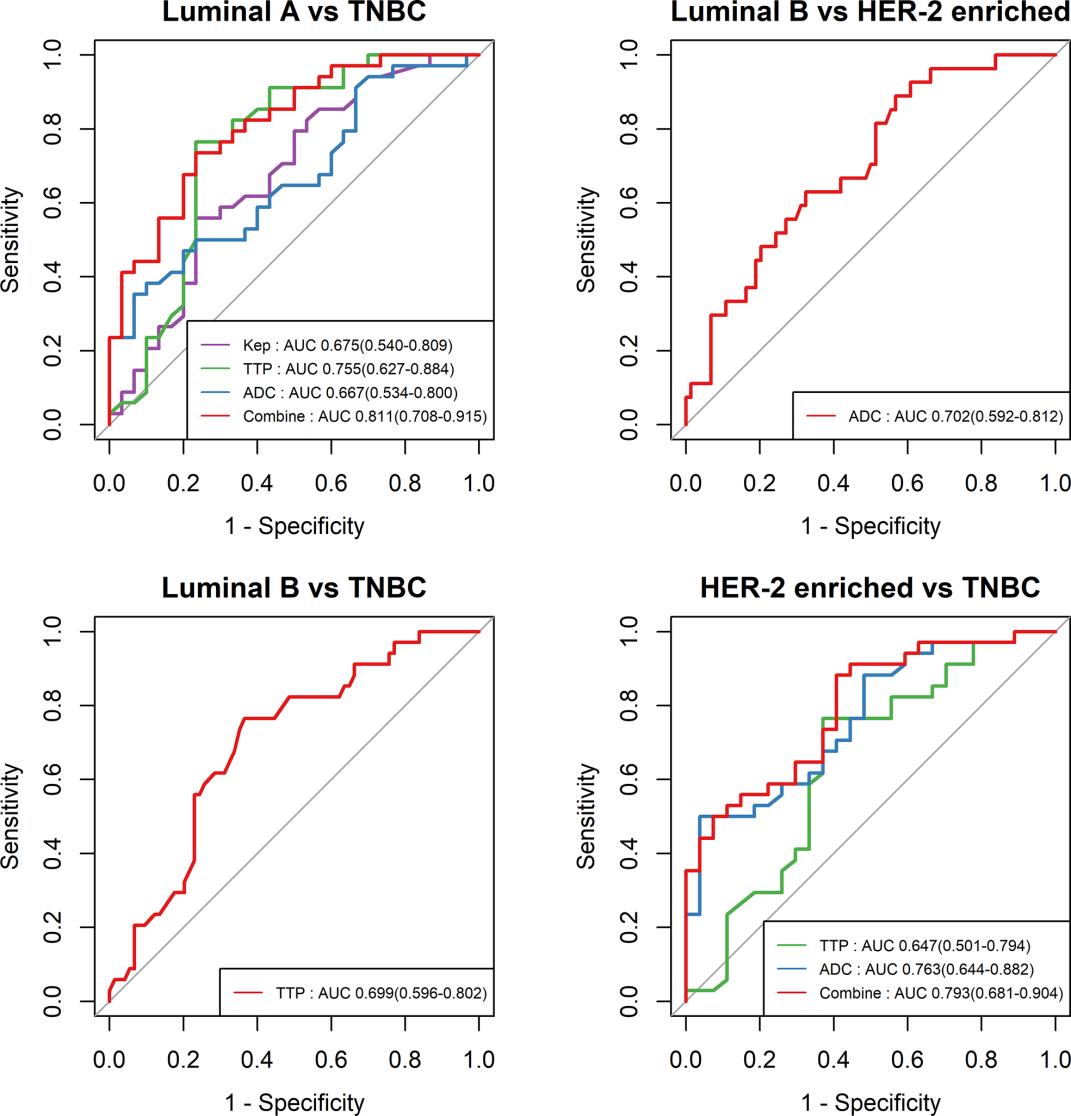
ROC curves of different parameters in the differential diagnosis between luminal A and TNBC, between luminal B and HER-2-enriched BC, between luminal B and TNBC, and between HER-2-enriched BC and TNBC.

The cutoff value, AUC, accuracy, sensitivity and specificity of these MR parameters with significant differences were further calculated using ROC curves (**Table 5** and **Figure 4**). In identifying TNBC and luminal A, the combination model of K_ep,_ TTP and ADC achieved the highest performance in the differential diagnosis, with an AUC of 0.811 (95% CI: 0.708–0.915). In identifying HER-2-enriched BC and TNBC, the combination model of ADC and TTP achieved the highest performance in the differential diagnosis, with an AUC of 0.793 (95% CI: 0.681–0.904).

**Table 5:** ROC curves of K_ep_, TTP and ADC in differential diagnosis.

		ROC analysis				
Identifying group	Parameters	Cutoff	AUC (95%CI)	Accuracy	Sensitivity	Specificity
Luminal A *vs.* TNBC	K_ep_	0.892	0.675 (0.540–0.809)	0.656	0.559	0.767
	TTP	0.582	0.755 (0.627–0.884)	0.766	0.765	0.767
	ADC	0.719	0.667 (0.534–0.800)	0.625	0.353	0.933
	Combined	0.527	0.811 (0.708–0.915)	0.750	0.735	0.767
Luminal B *vs.* HER-2-enriched	ADC	0.778	0.702 (0.592–0.812)	0.554	0.889	0.432
Luminal B *vs.* TNBC	TTP	0.583	0.699 (0.596–0.802)	0.676	0.765	0.635
HER-2-enriched *vs.* TNBC	TTP	0.582	0.647 (0.501–0.794)	0.705	0.765	0.630
	ADC	0.759	0.763 (0.644–0.882)	0.705	0.500	0.963
	Combined	0.405	0.793 (0.681–0.904)	0.754	0.882	0.593

Combined indicate the predicted model based on the combination of the parameters. ROC, Receiver operating characteristic; AUC, Area under the curve; CI, Confidence interval; TNBC, Triple-negative breast cancer; ADC, Apparent diffusion coefﬁcient.

## Discussion

This study explored the high-temporal resolution quantitative MRI parameters derived from CDT-VIBE and RS-EPI for the diagnosis of receptor status and molecular subtypes of BC. Our study showed that a lower K_ep_ value was independently associated with PR-positive status, and a higher TTP value was independently associated with ER- and PR-positive status. In addition, a lower TTP value and lower ADC value had stronger independent associations with TNBC than with Luminal A BC, while a lower ADC value had a stronger independent association with TNBC than with HER-2 enrichment. Furthermore, K_ep_, TTP and ADC permit the differential diagnosis between luminal A and TNBC, with the highest AUC of 0.811 (95% CI: 0.708–0.915), and compared with the results of Chang et al (19), our model has an higher AUC (0.811 *vs.* 0.636), with a higher accuracy (0.750 *vs.* 0.696) and specificity (0. 767 *vs.* 0.511). ADC and TTP permit the differential diagnosis between HER-2-enriched BC and TNBC, with the highest AUC of 0.793 (95% CI: 0.681–0.904).

In our study, the K_ep_ values were lower in hormone receptor-positive patients than in hormone receptor-negative patients, and further analysis revealed that a lower K_ep_ value was independently associated with PR-positive status. This finding is different from previous findings (20) that showed that K_ep_ derived from conventional DCE-MRI by using three-dimensional T1-weighted sequence axial scanning (a total of 6 phases,a total acquisition time beyond 6 min) was not associated with hormone receptor status. A potential explanation for CDT-VIBE improving the results may be as follows: The scan duration (<6 min) after injection of contrast medium has a significant impact on pharmacokinetic parameters (12), and CDT-VIBE could have improved the temporal resolution of each phase (a total of 34 phases) within the total acquisition time of 5 min 5 s. Therefore, our study may indicate that the K_ep_ derived from CDT-VIBE may be a more sensitive biomarker for predicting BC receptor status compared with the parameters of conventional DCE-MRI. In addition,K_ep_ was the highest in HER-2-enriched BC and the lowest in luminal A. This finding was consistent with the results of previous studies that indicated that more aggressive tumors have a higher K_ep_ value (21).

TTP is also closely related with angiogenesis. In this study, a higher TTP value was independently associated with hormone receptor-positive status. This finding is partly in line with previous findings that showed that a higher TTP value, derived from DCE-MRI by using a 3D fast spoiled gradient-echo sequence with parallel imaging acceleration, was correlated with hormone receptor-positive status (22); however, the trend remained significant in our study after applying the univariate and multivariate analysis. In addition, TTP was found to be significantly different between luminal A and TNBC, and a lower TTP (<0.582 min) had a stronger independent association with TNBC than with luminal A. In identifying luminal A and TNBC, TTP had a sensitivity of 0.766 and a specificity of 0.765. Because there is a lack of a single highly reliable parameter to identify luminal A and TNBC, the prediction model combining multiple parameters becomes a viable alternative. By incorporating K_ep_ and ADC into the prediction model, the overall predictive ability in the cohort was strong with an AUC of 0.811 (95% CI: 0.708–0.915). This finding was in excellent agreement with previous findings regarding the differentiation between luminal A and TNBC by using multiparametric MRI radiomics (23).

Diffusion imaging is a powerful technique to noninvasively measure microstructures and the ADC obtained is a quantitative measurement of water molecule diffusion (24). Bickel et al. proved ADC to be a valuable noninvasive quantitative biomarker for assessing BC invasiveness (25). Agreeing with previous studies (26–29), our results showed that a lower ADC was associated with higher tumor malignancy, especially in TNBC patients. This phenomenon may be attributed to the high cellular density and the restriction of water molecules in malignant tumors (30). A recent study further revealed that the features of DWI could reflect the intrinsic heterogeneous characteristics of molecular subtypes in BC (31). Our study further quantified the ADC derived from RS-EPI DWI and demonstrated that the ADC value was statistically significant in identifying HER-2-enriched BC and TNBC with an AUC of 0.763 (95% CI: 0.681–0.904). When TTP was incorporated into the prediction model, the AUCs achieved an approximate 30% increase.

There are some limitations of this study. First, although it is a relatively large cohort study with 165 patients, the inclusion of more cases in each subtype would make the results more reliable. Second, further comparisons of multiple modality imaging, such as ultrasound and mammography, would reveal a more comprehensive picture of breast cancer. Finally, previous studies have found that the combination of diffusion and enhanced imaging might improve the diagnostic performance in monitoring NAC (32). Therefore, it would be beneficial to apply our improved quantitative imaging methods to NAC in future clinical practice.

In conclusion, this study demonstrates that K_ep_ and TTP derived from DCE-MRI by using CDT-VIBE (i.e., high-temporal resolution quantitative MRI parameters) are independently associated with the hormone receptor status of BC. In addition, the K_ep_, TTP, and ADC values had stronger independent associations with TNBC than with luminal A and could differentiate TNBC from luminal A, with the highest AUC of 0.811 (95% CI: 0.708–0.915). Therefore, the high-temporal resolution quantitative parameters of K_ep_, TTP, and ADC appear to be useful noninvasive imaging biomarkers for predicting the receptor status and molecular subtypes of BC.

## Data Availability Statement

The data cohorts used and/or analyzed in the present study are available from the corresponding authors upon reasonable request.

## Ethics Statement

The studies involving human participants were reviewed and approved by Meizhou People’s Hospital. Written informed consent for participation was not required for this study in accordance with the national legislation and the institutional requirements. Written informed consent was not obtained from the individual(s) for the publication of any potentially identifiable images or data included in this article.

## Author Contributions

XGC and WF proposed the study concepts, and XFC, ZY, and ZD designed the study. Data acquisition was performed by FC. TZ and YL performed the statistical analysis. All author contributed to data analysis and interpretation. XFC and ZY were the major contributors and contributed equally in writing to manuscript. All authors contributed to the article and approved the submitted version.

## Funding

This study was supported in part by grants from the Natural Science Foundation of Guangdong Province (2018A030307057) and the Department of Education of Guangdong Province (2020KZDZX1085).

## Conflict of Interest

YL is a consultant for GE Healthcare.

The remaining authors declare that the research was conducted in the absence of any commercial or financial relationships that could be construed as a potential conflict of interest.

## Publisher’s Note

All claims expressed in this article are solely those of the authors and do not necessarily represent those of their affiliated organizations, or those of the publisher, the editors and the reviewers. Any product that may be evaluated in this article, or claim that may be made by its manufacturer, is not guaranteed or endorsed by the publisher.
